# Digitale Gesundheitsanwendungen (DiGA) im Spannungsfeld von Fortschritt und Kritik: Diskussionsbeitrag der Fachgruppe „Digital Health“ der Gesellschaft für Informatik e. V.

**DOI:** 10.1007/s00103-023-03804-2

**Published:** 2023-12-12

**Authors:** Hannes Schlieter, Maren Kählig, Emily Hickmann, Daniel Fürstenau, Ali Sunyaev, Peggy Richter, Rüdiger Breitschwerdt, Christian Thielscher, Martin Gersch, Wolfgang Maaß, Melanie Reuter-Oppermann, Lena Wiese

**Affiliations:** 1 Forschungsgruppe Digital Health, Fakultät Wirtschaftswissenschaften, Technische Universität Dresden, Dresden, Sachsen Deutschland; 2 Department of Business IT, IT University of Copenhagen, Kopenhagen, Dänemark; 3 Institute of Medical Informatics, Charité – Universitätsmedizin Berlin, Berlin, Deutschland; 4 Institut für Angewandte Informatik und Formale Beschreibungsverfahren, Karlsruher Institut für Technologie, Karlsruhe, Baden-Württemberg Deutschland; 5 Professur für Wirtschaftsinformatik und Medizinische Informatik, Wilhelm Büchner Hochschule, Darmstadt, Hessen Deutschland; 6 KompetenzCentrum für Medizinoekonomie, FOM Hochschule, Essen, Nordrhein-Westfalen Deutschland; 7 Fachbereich Wirtschaftswissenschaften, Freie Universität Berlin, Berlin, Deutschland; 8 Lehrstuhl für Betriebswirtschaftslehre, insbesondere Wirtschaftsinformatik, German Research Center for Artificial Intelligence Universität Saarland, Saarbrücken, Saarland Deutschland; 9 Faculty of Behavioural, Management and Social Sciences, Center for Healthcare Operations Improvement and Research, University of Twente, Enschede, Niederlande; 10 Fraunhofer Institute for Toxicology and Experimental Medicine ITEM, Leader Research Group Bioinformatics, Hannover, Niedersachsen Deutschland

**Keywords:** Gesundheitsapp, Medizinprodukt, Digitalisierung, Marktdurchdringung, Innovation, Health app, Medical device, Digitalisation, Market penetration, Innovation

## Abstract

Im Dezember 2019 wurden in Deutschland Digitale Gesundheitsanwendungen (DiGA) in die Regelversorgung aufgenommen und können somit durch die gesetzlichen Krankenkassen erstattet werden, um PatientInnen bei der Behandlung von Erkrankungen oder Beeinträchtigungen zu unterstützen. Inzwischen gibt es 48 DiGA (Stand: Oktober 2023) im Verzeichnis des Bundesinstituts für Arzneimittel und Medizinprodukte (BfArM), die vor allem in den Bereichen mentale Gesundheit, Hormone und Stoffwechsel sowie Muskeln, Knochen und Gelenke eingesetzt werden. In diesem Artikel beschreibt die Fachgruppe „Digital Health“ der Gesellschaft für Informatik e. V. (GI) die aktuellen Entwicklungen rund um die DiGA sowie das derzeitige Stimmungsbild zu Themen wie Nutzerzentrierung, Akzeptanz von PatientInnen und Behandelnden sowie Innovationspotenzial. Zusammenfassend haben DiGA in den letzten 3 Jahren eine positive Entwicklung in Form eines langsam steigenden Angebots verschiedener DiGA und Leistungsbereiche erfahren. Nichtsdestotrotz sind in einigen Bereichen noch erhebliche regulatorische Weichenstellungen notwendig, um DiGA langfristig in der Regelversorgung zu etablieren. Zentrale Herausforderungen bestehen u. a. in der Nutzerzentrierung oder in der nachhaltigen Verwendung der Anwendungen.

## Abstract (en)

Since December 2019, digital health applications (DiGA) have been included in standard care in Germany and are therefore reimbursed by the statutory health insurance funds to support patients in the treatment of diseases or impairments. There are 48 registered DiGA listed in the directory of the Federal Institute of Drugs and Medical Devices (BfArM), mainly in the areas of mental health; hormones and metabolism; and muscles, bones, and joints. In this article, the “Digital Health” specialist group of the German Informatics Society describes the current developments around DiGA as well as the current sentiment on topics such as user-centricity, patient and practitioner acceptance, and innovation potential. In summary, over the past three years, DiGA have experienced a positive development, characterized by a gradually increasing availability of various DiGA and coverage areas as well as prescription numbers. Nevertheless, significant regulatory adjustments are still required in some areas to establish DiGA as a well-established instrument in long-term routine healthcare. Key challenges include user-centeredness and the sustainable use of the applications.

Am 19.12.2019 nahm Deutschland, als eines der ersten Länder weltweit, Digitale Gesundheitsanwendungen (kurz: DiGA) in den Leistungskatalog der gesetzlichen Krankenkassen und damit in die Regelversorgung auf. Aufgrund dieser Entwicklung hat Deutschland eine internationale Vorreiterrolle auf diesem Gebiet eingenommen. Im Folgenden wird der gegenwärtige Stand der Innovation DiGA in verschiedenen Kategorien dargelegt, einschließlich positiver Entwicklungen sowie einer kritischen Auseinandersetzung. Diese Kategorien umfassen:

- die nutzerzentrierte Gestaltung von DiGA,
- die Integration von DiGA in die Versorgung sowie
- die Akzeptanz von DiGA bei PatientInnen und Behandlern.

## Hintergrund und Status quo

Digitale Gesundheitsanwendungen sind „digitale Medizinprodukte niedriger Risikoklasse, die die Versicherten etwa bei der Behandlung von Erkrankungen oder dem Ausgleich von Beeinträchtigungen unterstützen können“ [1]. Hierbei handelt es sich um sogenannte Apps, also Softwareanwendungen, die auf dem Smartphone oder einem Tablet genutzt werden können, aber auch um webbasierte Anwendungen, die über einen Internetbrowser auf einem PC oder Laptop bedient werden [2]. DiGA folgen dem Konzept „App auf Rezept“, so könnten sie, ähnlich wie Arzneimittel, vorbeugend, therapierend, lindernd oder verzögernd verordnet werden. Der aktuelle Referentenentwurf des Bundesministeriums für Gesundheit (BMG) vom 15.06.2023 zum Digitalgesetz sieht zudem eine Ausweitung auf die Risikoklasse 2b vor, was auch den Einsatz von DiGA bei komplexeren Erkrankungen ermöglichen kann.

Ein Beispiel ist die DiGA Kalmeda® der Firma mynoise Gesellschaft mbH aus Duisburg, welche Ende September 2020 als erste Anwendung im Verzeichnis aufgenommen wurde. Auf Basis einer kognitiven Verhaltenstherapie, der einzigen leitlinienbasierten Behandlungsform bei chronischem Tinnitus, unterstützt die App PatientInnen dabei, besser mit den Ohrgeräuschen zurechtzukommen [3]. Ein anderes Beispiel ist inVirto® der Firma Sympatient GmbH aus Hamburg, eine DiGA, die Personen mit Angststörungen hilft, mittels Expositionstherapie der Krankheit zu begegnen und Ängste abzubauen. In diesem Fall wird die DiGA zusammen mit einer VR-Brille verwendet [4]. Anhand der verhaltenstherapeutischen Ansätze, die beispielsweise dabei unterstützen, gesunde Gewohnheiten zu etablieren, soll der alltägliche Umgang mit der Erkrankung positiv beeinflusst werden.

Deutschland ist eines der ersten Länder, in dem ein institutionell geregeltes Zulassungsverfahren für mobile Gesundheitsanwendungen eingeführt wurde [5]. Im sogenannten Fast-Track-Verfahren des Bundesinstituts für Arzneimittel und Medizinprodukte (BfArM) wird bereits innerhalb von 3 Monaten über die Zulassung und die damit mögliche Abrechnung mit allen deutschen Krankenkassen entschieden [6].

Aktuell (07/2023) sind 48 DiGA im Verzeichnis des Bundesinstituts für Arzneimittel und Medizinprodukte (BfArM) enthalten [4]. Für die Aufnahme ins Verzeichnis müssen Hersteller anhand einer vergleichenden Studie den Nachweis eines positiven Versorgungseffekts für eine bestimmte Patientengruppe in mindestens einem der vordefinierten Endpunkte in den Kategorien (i) medizinischer Nutzen oder (ii) patientenrelevante Struktur- und Verfahrensverbesserungen erbringen. Bei einem bereits vorhandenen Nachweis kann die direkte Aufnahme in das Verzeichnis beantragt werden. Ist noch kein Nachweis vorhanden, kann nur eine vorläufige Aufnahme beantragt werden anhand einer systematischen Datenauswertung, welche plausibel belegt, dass sich im Rahmen der max. 24-monatigen Erprobungsphase die erforderliche Evidenz durch eine Studie nachweisen lässt. Ansonsten wird die DiGA wieder aus dem Verzeichnis gestrichen [6]. Mit dem benötigten Nachweis kann dann der Antrag auf eine dauerhafte Aufnahme gestellt werden.

Bei den „patientenrelevanten Struktur- und Verfahrensverbesserungen“ handelt es sich um ein neues ins deutsche Sozialgesetzbuch V (SGB V) eingeführtes Konzept. Hierunter fallen zum Beispiel eine verbesserte Adhärenz, Patientensicherheit oder Gesundheitskompetenz [6]. Der zu erbringende Nachweis erfolgt meist im Vergleich zu einer Nichtanwendung der Intervention und wird in der Regel anhand einer randomisierten kontrollierten Studie erbracht [6]. In dieser Erprobungsphase befinden sich derzeit 29 der 47 im Verzeichnis gelisteten DiGA [4].

Egal, ob eine vorläufige oder direkte Aufnahme ins Verzeichnis vorliegt – der vom Hersteller vorgeschlagene Preis der Leistung wird für 12 Monate erstattet. Danach löst der zwischen dem Hersteller und dem Spitzenverband Bund der Krankenkassen (GKV-SV) verhandelte Preis den zuvor veranschlagten Herstellerpreis ab. Der Referentenentwurf zum Digitalgesetz sieht hier zukünftig eine Preisgestaltung vor, die sich an anwendungsbegleiteten Erfolgskriterien anlehnt [7]. Dies soll mehr Anreize für eine nachhaltige Etablierung am Markt und mehr Transparenz bei der Preisbildung generieren.

Bis Juli 2023 waren insgesamt 171 Anträge auf Zulassung einer DiGA beim BfArM eingegangen. Zahlreiche Anträge wurden wieder zurückgezogen. Die meisten DiGA veranschlagen eine Nutzungsdauer von 3 Monaten. In diesem Zeitraum kosten DiGA durchschnittlich 526 €/PatientIn, bei einer Preisspanne zwischen 119 € und 2077,40 € [8].

Während zunächst von einer verhaltenen Verschreibungspraxis berichtet wurde, ergaben sich im letzten Jahr steigende Verschreibungszahlen, die aus der zunehmenden Anzahl eingelöster Freischaltecodes für DiGA ersichtlich sind (1. Halbjahr 2021 vs. 1. Halbjahr 2022, Tab. 1). Neuere Bilanzen zeigen jedoch ein eher stagnierendes Verschreibungsbild [9]. Die tatsächliche Marktgröße und das weitere Wachstum des DiGA-Marktes lassen sich noch schwer prognostizieren. Diese hängen auch wesentlich von den regulatorischen Weichenstellungen, den steigenden Hürden beim Markteintritt für potenzielle DiGA-Anbieter (z. B. in Form der neu zu erbringenden Datenschutz-Zertifizierung nach Datenschutz-Grundverordnung – DSGVO; [10, 11]) sowie der Akzeptanz durch PatientInnen und Verordnende ab.

**Table 1:** 

Krankenkasse	Erstes Halbjahr 2021	Erstes Halbjahr 2022	% Wachstumsrate 2021–2022	Anzahl Versicherte gesamt (Stand 07.2022)
AOK	4920	18.078	267	27.185.248
TK	6082	13.993	130	11.004.471
Barmer	3320	7598	129	8.707.552
Gesamt	14.322	39.669	177	46.897.271

Dieser Diskussionsbeitrag bietet eine kritische Übersicht über die aktuellen Entwicklungen von DiGA. Er präsentiert ein Stimmungsbild zu zentralen Themen der DiGA-Entwicklung wie nutzerzentriertes Design, Akzeptanz und Innovationspotenzial. Er zeigt Herausforderungen und Chancen von DiGA und potenzielle Hindernisse für die weitere Integration in die Gesundheitsversorgung.

Eine entscheidende Komponente in diesem Kontext sind die effektive und nutzerfreundliche Gestaltung ebenso wie die Berücksichtigung ethischer und datenschutzrechtlicher Aspekte. Einen Überblick über die wichtigsten Diskussions- und Kritikpunkte bietet die Infobox 1.

## Nutzerzentrierte Gestaltung von DiGA

Eine nutzerzentrierte Entwicklung und Gestaltung ist das Fundament etablierter digitaler Innovationen [12]. So konnte nachgewiesen werden, dass neben traditionellen klinischen Parametern als Wirksamkeitsmaß einer digitalen Intervention auch die Akzeptanz, die subjektiv wahrgenommene Nützlichkeit sowie die Bedienbarkeit für PatientInnen wichtige Faktoren für eine langfristige Nutzung darstellen [13, 14]. Wenn diese zu gering ausgeprägt sind, kann es also trotz eines therapeutischen Wirksamkeitsnachweises zu geringer Adhärenz seitens der PatientInnen kommen.

Die von DiGA-Anbietern entwickelten Lösungen haben grundsätzlich das Ziel, die Gesundheitsversorgung ganzheitlich, patientenzentriert und sinnvoll zu verbessern. Um die Finanzierung der DiGA zu sichern, stehen jedoch innerhalb der Konzeptionsphase meist die Hürden des Fast-Track-Zulassungsverfahrens im Fokus, wie beispielsweise der Nachweis des Versorgungseffekts oder technische Anforderungen. Der Effekt des sich daraus ergebenden finanziellen Drucks für die Hersteller wird noch verschärft, wenn einige Fördergeber gezielt die Entwicklung von DiGA aus ihren Förderprogrammen ausschließen (z. B. Versorgungsforschung des Gemeinsamen Bundesausschuss), wodurch DiGA-Hersteller weniger Möglichkeiten haben, eine Finanzierung ihrer Vorhaben zu realisieren.

„Der Weg zu einer DiGA ist für die vielen Startups, mit denen wir täglich zu tun haben, nicht gerade leicht. Die ÄrztInnen wollen ihre ausgereiften und teilweise sehr innovativen Therapieverfahren im Jahr vielen 1000 bis 10.000 PatientInnen statt nur wenigen 100 PatientInnen in der eigenen Praxis zur Verfügung stellen. Dies scheint über den Weg der DiGA ein attraktiver Weg zu sein. Aufgrund der hohen Gesamtkosten einer DiGA-Entwicklung (Konzeption, technische Entwicklung, Studiendesign, Studiendurchführung, Vermarktung, etc.) und den aktuell wenigen Förderungen bzw. Unterstützungen, wird es den GründerInnen leider nicht gerade leicht gemacht und viele scheitern an der Finanzierung. Damit werden diverse digitale Anwendungen mit positiven Versorgungseffekten für die PatientInnen leider nicht realisiert und dem Gesundheitssystem entgehen spannende DiGAs“ (Malte Bornholdt, Geschäftsführer der Bornholdt Lee GmbH, 20.07.2023).

Da eine DiGA ein Medizinprodukt ist, unterliegt sie zudem besonderen Werbebeschränkungen, was weitere Limitationen von Finanzierungs- und Absatzmöglichkeiten schafft. So ist eine zusätzliche öffentlichkeitswirksame Werbung für DiGA nicht statthaft bzw. nur unter strengen Auflagen möglich, sodass hauptsächlich das DiGA-Verzeichnis als Präsentationsplattform bleibt. Ein aktives Anpreisen von zusätzlichen Leistungen etc. wird untersagt, wobei das Vorhalten solcher Angebote grundsätzlich nicht verboten ist. So könnte, neben dem zentralen Versorgungscharakter, die Möglichkeit von In-App-Käufen zu zusätzlichen Einnahmen beitragen. Beispiel hierfür sind zusätzliche Funktionen oder Prämienvariationen, welche innerhalb der Anwendung durch die Nutzer zusätzlich erworben werden können [6]. Dies könnte zukünftige und zwingend erforderliche Entwicklungskosten kompensieren und sogar die nachhaltige Nutzung im Rahmen der beworbenen In-App-Käufe fördern, ohne sich direkt auf die Gesundheitskosten auszuwirken.

Auf dem Weg zur Zulassung ist zudem die Nutzerfreundlichkeit nur eines von zahlreichen Kriterien im Zulassungsprozess, welche in Form von Checklisten durch die Hersteller geprüft werden. Häufig entsteht so der Eindruck, dass eine echte Nutzerzentrierung noch nicht Kernbestandteil der Entwicklung digitaler Lösungen ist. Um eine nachhaltige Nutzerzentrierung zu erreichen, bietet sich die Möglichkeit einer engen Zusammenarbeit mit den Patientenvertretungen und den verordnenden Professionen an. So können bereits im frühen Konzeptionsstadium einer DiGA Benutzerfreundlichkeit und Nutzerpräferenzen untersucht und berücksichtigt werden [13].

Zum aktuellen Zeitpunkt sind DiGA-Hersteller durch § 18 der Digitale-Gesundheitsanwendungen-Verordnung (DiGAV) bei der Veränderung wesentlicher Merkmale ihrer DiGA eingeschränkt. Zwar wird genau definiert, was eine „wesentliche Veränderung“ bedeutet, wie beispielsweise eine Änderung mit Einfluss auf die Datensicherheit, Funktionstauglichkeit und den positiven Versorgungseffekt, allerdings ist es in jedem Fall angebracht, Veränderungen der DiGA anzuzeigen. So können unter Umständen bereits Änderungen der Usability Einfluss auf den Versorgungseffekt haben.

Die Bewertung der Änderungen anhand der festgelegten Aufnahmekriterien durch das BfArM entscheidet letztlich über das weitere Bestehen oder die Streichung der App aus dem Verzeichnis. Daher geht mit Veränderungen der Anwendung, z. B. aufgrund von später erkannten oder geänderten Bedürfnissen von PatientInnen, immer auch die Gefahr einer Streichung für die DiGA-Hersteller einher [6].

Weiterhin zeigt sich, dass das Kriterium „patientenrelevante Struktur- und Verfahrensverbesserung“ als primärer Wirkungsnachweis (sogenannter primärer Endpunkt), als eigentlich zentrales Wirkungsinstrument von DiGA, von kaum einer App abgebildet wird. Hier sehen die AutorInnen einen wesentlichen Kritikpunkt an den bestehenden DiGA. Der momentane Schwerpunkt bei der Evaluation von DiGA anhand typischer klinischer Endpunkte beeinträchtig die Entwicklung von DiGA, die vor allem auf patientenrelevante Strukturverbesserungen zielen. Das sind zum Beispiel DiGA, die auf Themen wie Patienten-Empowerment setzen. Klinische Endpunkte werden vor allem deshalb gewählt, weil es an der Verfügbarkeit akzeptierter Messinstrumente für Strukturverbesserungen mangelt und die Folgen für die Preisverhandlung unklar sind [15].

Ist der Genehmigungsprozess gemeistert, muss die DiGA, um sich zu etablieren, beworben, verschrieben und letztendlich auch genutzt werden [16]. Eine nachhaltige Nutzung erfordert hierbei immer auch ein gewisses Maß an digitaler Gesundheitskompetenz der NutzerInnen. So sollte an dieser Stelle hervorgehoben werden, dass den Kostenträgern laut § 20k SGB V eine besondere Rolle bei Aufbau und Förderung benötigter digitaler Kompetenzen zukommt. Diese wichtige Lotsenfunktion kann einen wesentlichen Beitrag bei der Implementierung und Marktdurchdringung von DiGA leisten.

Ein vielversprechendes Potenzial von DiGA bietet außerdem die Generierung von „Real-World Data“, um DiGA-Daten longitudinal zu tracken und so ein „lernendes System“ zu schaffen. Damit könnten einerseits Aussagen über die Wirkung von DiGA in der Anwendung bei PatientInnen getroffen und andererseits die generierten Daten zu Forschungszwecken und zur Weiterentwicklung der jeweiligen DiGA eingesetzt werden [17]. Diese Möglichkeiten werden bisher jedoch in zu geringem Maß genutzt. Bestehende Ansätze zum Austausch von DiGA-generierten Daten mit Dritten (insbesondere auch ForscherInnen aus der Informatik) befinden sich noch in der Entwicklung. Um hierbei die Effizienz der Wiederverwendung der Forschungsdaten sicherzustellen, sollten die FAIR-Prinzipien (Findability, Accessibility, Interoperability und Reusability^1^) beachtet werden [18]. Außerdem kann durch das Real World Evidence-Studienprotokoll (RWE) auf eine einheitliche Vorlage für reproduzierbare „Real-World-Evidence“-Studien zurückgegriffen werden [19].

## DiGA in der integrierten Versorgung

Digitale Gesundheitsanwendungen bieten eigentlich die Möglichkeit und den indikationsbezogenen Mehrwert, PatientInnen entlang ihrer gesamten Versorgungskette zu begleiten und durch ein individuelles Behandlungsregime zu unterstützen [20]. Durch die geforderte starke Zuspitzung des Interventionszweckes finden sich aktuelle DiGA jedoch vorwiegend im ambulanten Sektor wieder. So auch Prof. Fürstenau, Sprecher der Fachgruppe „Digital Health“ der Gesellschaft für Informatik e. V., beim GI-WebTalk am 01.09.2022:

„In der konkreten Ausgestaltung des DVG gibt es sehr viele Punkte, an denen man ansetzen und wo man die Regulatorik auch weiterentwickeln könnte, damit wir dahin kommen, dass wir zukünftig ein stärker vernetztes und besser aufeinander abgestimmtes Versorgungssystem erreichen. Heutzutage sind DiGAs sehr stark als Stand-alone-Therapien gedacht.“

Seit März 2022 können im Rahmen des Entlassmanagements, im klinischen Kontext, DiGA als Unterstützung verordnet werden. Ziel ist, Behandlungserfolge zu sichern, indem der Übergang von der stationären zur ambulanten Versorgung verbessert wird. Zum Einsatz von DiGA im Entlassmanagement liegen derzeit allerdings aufgrund der mangelnden Umsetzung in der Praxis nur wenige Studienergebnisse vor [21]. Vor allem aber stellen das Fehlen der Entlassmanagement-DiGA im Rahmenvertrag der Selbstverwaltungspartner [22] und die derzeit nur eingeschränkte Integration von DiGA in andere Systeme [23] einen wesentlichen verzögernden Faktor dar. Obwohl das „Medizinische Informationsobjekte (MIO) DiGA Toolkit“ der Kassenärztlichen Bundesvereinigung (KBV) geschaffen wurde, um eine Kommunikation zwischen DiGA und anderen Systemen zu ermöglichen, gibt es noch immer Probleme bei der Darstellung der MIO in der Praxisverwaltungssoftware [24]. In diesem Zusammenhang ist die DiGA-Regulatorik zu prüfen sowie zielgerichtet weiterzuentwickeln. Auch ist eine enge Zusammenarbeit zwischen DiGA-Anbietern, Regulatorik, Kassenärztliche Bundesvereinigung (KBV) sowie Anbietern von Praxisverwaltungssystemen (PVS) notwendig, um eine nutzerzentrierte Umsetzung zu gewährleisten. Das erste MIO-Toolkit ist seit 01.01.2023 gültig und soll verpflichtend von den Herstellern umgesetzt werden [25]. Grundsätzlich muss hier auf allen Ebenen die Interoperabilität als Verpflichtung gesehen werden, um einem „Information Blocking“ vorzubeugen und dieses in den bestehenden Strukturen abzubauen.

In der Zukunft sollte die Verschränkung von DiGA im integrierten Versorgungsprozess noch weitergedacht werden:

„Im deutschen Gesundheitswesen braucht es endlich in der Breite indikationsspezifisch und sich am Patientennutzen orientierende Anwendungen, welche den Therapieplan digitalisieren aber auch Interaktions‑, Informations- und Selbstmanagement-Funktionen bereithalten. Solche Anwendungen haben den Charakter von ‚Master-Apps‘, die neben bekannten Dingen wie Arzneimittelinformationen weitere Mehrwertfunktionen für den jeweiligen Versorgungskontext bereithalten. So bieten sich nach der Entlassung aus dem Krankenhaus bspw. Text-Chat und Video-Chat mit Behandlern (Stichwort: ‚KIM/TIM^2^‘), die PRO-Erfassung (Patient-Reported Outcomes), oder Therapiekomponenten (DiGA, Patient-Education) an. In einer integrierten App sollten diese Funktionen verfügbar sein und für eine integrierte Therapieerfahrung sorgen“ (Dr. Hannes Schlieter, Sprecher der GI-Fachgruppe Digital Health, 04.09.2022).

Einige DiGA-Anbieter ermöglichen PatientInnen aus der Anwendung heraus ein PDF aus ihren Daten (z. B. persönliche Daten, Trainings‑, Schmerz- und Funktionsverlauf) zu erstellen, welches wiederum den Behandelnden als Therapiebericht vorgelegt werden soll [26]. Ziel sollte hier jedoch die Möglichkeit einer digitalen Übertragung sein, um ressourcenintensive Zwischenschritte, wie das Übernehmen der Dokumente als Scan in die Patientenakte, abzubauen. Dabei ist zu wünschen, dass der Gesetzgeber DiGA nicht nur als zusätzliches Instrument sieht, sondern als festen Bestandteil von Versorgungsketten (auch im Zusammenhang mit der Verordnung als E‑Rezept). DiGA sollten aber auch insgesamt überdacht und neu gestaltet werden, wie dies z. B. im BMG-geförderten Projekt DIGIOP^3^ in Ansätzen vorgedacht wurde [27].

## Akzeptanz der PatientInnen und Behandelnden

Von den in Psychotherapie und Medizin verordneten DiGA wird ein Großteil (fast 90 % [28]) von Ärztinnen und Ärzten aus der Allgemeinmedizin, gefolgt von Hals-Nasen-Ohrenheilkunde und Orthopädie verschrieben [22]. Ähnlich der Anwendung eines Arzneimittels ist die Nutzung einer DiGA direkt von dem Verordnungsverhalten der PsychotherapeutInnen sowie Ärztinnen und Ärzten abhängig. Bekanntlich gibt es viele verschiedene Faktoren und Einflüsse, die dieses Verhalten beeinflussen können (siehe z. B. die „Theorie des geplanten Verhaltens“ [29]). Unter anderem spielt die Akzeptanz der MedizinerInnen, also der Grad der Überzeugung, ob sich die Verordnung der DiGA positiv auf das Wohl der PatientInnen auswirken wird, eine wichtige Rolle. Genau diese Akzeptanz ist in Bezug auf DiGA momentan noch ausbaufähig [30].

Eine Ursache für die geringe Akzeptanz bei Behandelnden könnte sein, dass sich einige der gelisteten DiGA in der Erprobungsphase befinden und daher noch keinen nachgewiesenen positiven Versorgungseffekt aufweisen [4]. Bei einer Befragung der Stiftung Gesundheit gaben 66 % der befragten MedizinerInnen an, dass klinische Evidenz ihre Akzeptanz steigert. Umgekehrt kann fehlende Evidenz ein großes Hemmnis für die Verschreibung darstellen [31].

Etwa 40 % der MedizinerInnen sehen in ihrer begrenzten Vertrautheit mit der digitalen Innovation einen Faktor, der die Akzeptanz negativ beeinflusst. Eine Rolle spielen hierbei auch der aktuell als kompliziert angesehene DiGA-Aktivierungsprozess (34 %), unzureichendes Informationsmaterial (32 %) und fehlende Möglichkeiten, eine Anwendung zu testen (47 %). Die Befragten wünschten sich mehr Schulungen zu DiGA sowie Testzugänge [31]. Diese müssen von den Leistungserbringenden allerdings auch wahrgenommen werden.

„Aktuell scheinen die meisten Kolleginnen und Kollegen DiGA als etwas zu sehen, das für manche Patienten vielleicht nützlich, aber nicht unbedingt erforderlich ist“ (Prof. Dr. med. C. Thielscher, Informatiker, Arzt und Wirtschaftswissenschaftler).

Hinzu kommt das teils undurchsichtige Genehmigungsverfahren der DiGA-Kostenträger bei Verordnungen. MedizinerInnen und PatientInnen selbst ist häufig unklar, warum eine DiGA bestimmten PatientInnen genehmigt wird und anderen nicht. Dem versucht nun der Gesetzgeber mit dem neuen Referentenentwurf zum Digitalgesetz nachzukommen und will die Kostenträger verpflichten, einheitliche Regelungen zur Genehmigung oder Ablehnung einer DiGA zu schaffen. Dies kann mehr Sicherheit aufseiten der DiGA-Hersteller, der verordnenden Leistungserbringer und PatientInnen schaffen.

Auch die hohe Sensibilität der Daten könnte zusätzliche Zurückhaltung hervorrufen, sowohl auf der Seite der Behandelnden als auch bei PatientInnen. Bereits einzelne Datenschutzverletzungen können das allgemeine Misstrauen gegenüber dem Schutz von Gesundheitsdaten verstärken (bspw. im Fall der Gesundheits-App Ada im Jahr 2019; [32]). In diesem Zusammenhang wird die Regulatorik zum Datenschutz bei DiGA allerdings stetig weiterentwickelt. Neben den bereits bestehenden Regelungen zum Nachweis eines Informationssicherheitsmanagements, inklusive Penetrationstest, bei welchem strukturiert durchgeführte Angriffe auf das IT-System mögliche Schwachstellen identifizieren, werden weitere verpflichtende Nachweise fällig [33]. So wird laut § 7 Abs. 3 DiGAV ab Januar 2024 ein Datensicherheitszertifikat des Bundesamts für Sicherheit in der Informationstechnik und ab August 2024 die Vorlage eines DSGVO-Zertifikats verpflichtend sein.

Beim Thema Datenschutz sollte auch bedacht werden, dass für die Weiterentwicklung von Diagnostik und Therapie eine datenschutzkonforme Weitergabe von personenbezogenen Daten von großem Wert ist. Bei einer verlässlichen Anonymisierung der Daten ist auch ein Großteil der Deutschen bereit Gesundheitsdaten zur Verfügung zu stellen [34]. Hier gilt es, standardisierte und transparente Prozesse zur Datenweiterleitung sicherzustellen. Wenn ein datenschutzkonformer Austausch von Daten gewährleistet ist, besteht aber immer noch Unsicherheit darüber, wer die Daten analysiert und wie die Ergebnisse weitergegeben werden. Um sowohl strukturelle als auch inhaltliche Aspekte transparent zu definieren und auszuführen, sind Datenverträge erforderlich, die von unabhängigen Dritten abgewickelt werden. Die GAIA‑X^4^-Architektur sieht solche Datenverträge grundsätzlich vor [35, 36].

Auffällig ist zudem, dass, obwohl vollständig ins Verzeichnis aufgenommene DiGA eine belastbare Evidenz aufweisen, diese trotzdem nicht von den medizinischen Leitlinien berücksichtigt werden. So empfiehlt die Arbeitsgemeinschaft der Wissenschaftlichen Medizinischen Fachgesellschaften e. V (AWMF) zwar die Berücksichtigung von Leitlinien in den DiGA, jedoch nicht die Anwendung von DiGA in den Leitlinien. Bemühungen diesbezüglich durch die AWMF sollten zukünftig forciert und zielgerichtet mit den Aktivitäten des Interop-Council verbunden werden. Dieses Spannungsfeld zwischen einer DiGA, welche sich an medizinischen Leitlinien ausrichtet, jedoch nicht von eben diesen Leitlinien empfohlen wird, gilt es zu überwinden und den gemeinsamen Innovationscharakter der medizinischen Fachgesellschaften und der Informatik zu stärken [37].

## Entwicklungspotenzial von DiGA

Obwohl DiGA seit 3 Jahren auf dem Markt sind und bereits einige Erfolge verzeichnen konnten, befinden sie sich immer noch in einem Entwicklungsstadium, was zwangsläufig mit einigen Defiziten einhergeht. Eine der größten Herausforderungen besteht darin, DiGA als digitales Artefakt zu verstehen und damit umzugehen, einschließlich der Risiken und des Entwicklungspotenzials. Wichtig ist hierbei, die Evolution der Anwendungen zu erleichtern und gleichzeitig die Sicherheit der NutzerInnen zu gewährleisten. Zum Beispiel könnten Nutzerfeedbacks besser berücksichtigt oder die Aufnahme neuer Funktionalitäten in die Produkte ermöglicht werden. So präsentieren sich die Wirkmechanismen von DiGA als wesentlicher Ansatzpunkt für die zukünftige Forschung.

Neben digitalen verhaltenstherapeutischen Angeboten und Virtual Reality ist beispielsweise Gamification (d. h. der Einsatz von Elementen des Game-Designs in spielfremden Kontexten) ein vielversprechender Ansatz, um die Nutzung von DiGA nachhaltig zu fördern. Dies kann unter anderem mit Quiz-ähnlichen Attributen gestaltet werden, bei welchen Punkte oder Ähnliches gesammelt werden. Gleichzeitig mangelt es jedoch noch an tiefgründigem Wissen und Erfahrungen über mögliche Langzeiteffekte und Wirkungsweisen spezifischer Game-Design-Elemente sowie über Potenziale der Personalisierung von Gamification.

Hinzu kommen beispielsweise Ansätze, therapeutische Bestandteile der DiGA als App in Plattformmodelle einzubetten [38]. Das heißt, DiGA nutzen Dienste und Infrastruktur einer Plattform, womit auch die Interaktion mit anderen DiGA ermöglicht wird. Ebenso könnte damit die Time-to-Market von DiGA drastisch reduziert werden. Die aktuelle Forschung zeigt jedoch: Um den wirklichen Nutzen von Plattformen-basierten Systemen zu heben, sollten das Meinungsbild und die Ansprüche der wichtigsten Interessengruppen bekannt sein. Dies kann beispielsweise in Produktlinienmodelle münden oder zu einer Art Baukasten für DiGA führen. Diese Vorteile sollten als Gestaltungsgrundlage der Plattformen berücksichtigt werden.

Daten, die durch DiGA gewonnen werden können, stellen zudem ein besonderes Wertgenerierungspotenzial für die medizinische Forschung dar. Die geplante EU-Verordnung des „European Health Data Space“ (EHDS: EU 2022; [39]) eröffnet eine vielversprechende Perspektive für derartige föderierte Datenräume in Europa. DiGA-Daten könnten durch die Integration in den EHDS eine wichtige Rolle bei der Entwicklung von umfassenden, datenbasierten Gesundheitslösungen spielen.

Auch der vermehrte Einsatz von künstlicher Intelligenz (KI) im Gesundheitswesen im Allgemeinen und DiGA im Speziellen macht zunehmende Forschungsanstrengungen im Hinblick auf deren Vertrauenswürdigkeit erforderlich [40]. Dies beinhaltet unter anderem die Erforschung von Ansätzen der erklärbaren KI (engl. Explainable AI) im DiGA-Kontext sowie Forschung zur Verantwortlichkeit und Zertifizierung KI-basierter DiGA. In diesem Zusammenhang bedarf es insbesondere mehr Forschung zur Dezentralität von DiGA, zum Beispiel zu dezentralem Daten- und Rechtemanagement mithilfe von emergenten Internet-basierten Technologien, wie z. B. Blockchain [41].

Aber auch der regulatorische Verordnungsprozess im Hinblick auf die Digitalisierungsstrategie lässt Potenzial ungenutzt. So könnte die Unsicherheit über den Verordnungsprozess beispielsweise durch das E‑Rezept in der Versorgung aufgelöst werden und DiGA stärker als sinnvolle Innovation in die digitale Gesundheitsversorgung aufgenommen werden.

Trotz der sehr kritischen Bewertung des aktuellen Umsetzungsstandes der Innovation DiGA können wir auf eine positive Entwicklung in den letzten Monaten zurückblicken. So zeigt sich, dass neben der DiGA auch andere digitale Versorgungsformen, wie die Innovationsfondsprojekte, Erfolge bei der Etablierung in der Regelversorgung verzeichnen und zunehmend an Akzeptanz gewinnen.

So resümiert die Deutschen Gesellschaft für Telemedizin (DGTelemed) vorsichtig optimistisch über bereits erfolgreich umgesetzte digitale Leuchtturmprojekte. Handlungsbedarf, und dieser kann auf alle digitalen Innovationen übertragen werden, wird weiterhin bei der Umsetzungsgeschwindigkeit und Transparenz auf Bundesebene gesehen [42]. Die Digitalstrategie des BMG versucht zukünftig genau hier anzusetzen und nimmt DiGA als Teil der hybriden Versorgungsprozesse innerhalb der Prozesstransformation auf. Hierdurch sollen langfristig digitale Prozesse helfen DiGA am Markt zu fördern und das volle Versorgungspotenzial patientenzentriert auszuschöpfen [43].

## Fazit

DiGA können einen großen Einfluss auf den deutschen und internationalen Gesundheitsmarkt haben. Mit der Aufnahme von DiGA in die Regelversorgung und dem bereits nachweisbaren Versorgungsnutzen sollten nun Maßnahmen, um DiGA von einem Nischenprodukt zu einem festen Bestandteil der Regelversorgung zu machen, weiter vorangetrieben werden. Die Digital-Health-Forschung und die Informatik sind hierbei aufgefordert, für Technologien den Weg zu ebnen, die bedarfsgerecht, wirksam und nutzerfreundlich sind und dabei ethische und datenschutzrechtliche Fragen berücksichtigen. Die Markenkernwerte deutscher DiGA sollten Offenheit, Interoperabilität und Wissenschaftlichkeit sein.

### Infobox Digitale Gesundheitsanwendungen – Diskussions- und Kritikpunkte in Bezug auf das bisherige DiGA-Verfahren

Konzeption von DiGA

- Eine nutzerzentrierte Entwicklung zur Förderung von Akzeptanz, Nützlichkeit und Benutzerfreundlichkeit wird in einem größeren Umfang benötigt.
- Bisher werden patientenrelevante Struktur- und Verfahrensverbesserungen der DiGA zu wenig in den Blick genommen.

Zulassungsprozess von DiGA

- Es besteht ein zu hoher finanzieller Druck bei der Entwicklung von DiGA durch wenige Finanzierungsmöglichkeiten und hohe Zulassungsvoraussetzungen.
- Werbemöglichkeiten und Optionen zusätzlicher Einnahmen für Hersteller sind sehr beschränkt.
- Wesentliche Änderungen der Anwendung gehen mit dem Risiko der Streichung aus dem DiGA-Verzeichnis einher.

Integration in den Versorgungsprozess

- Es bestehen Herausforderungen bei der Integration von DiGA in Versorgungssysteme aufgrund fehlender Interoperabilität.
- Bei MedizinerInnen und TherapeutInnen besteht aktuell noch eine geringe Akzeptanz aufgrund der mangelnden klinischen Evidenz, fehlender eigener digitaler Gesundheitskompetenz und aufgrund des komplizierten Verordnungsprozesses.
- Bedenken existieren bezüglich Datenschutz und Unsicherheiten bei der Datenanalyse und -weitergabe.
- Der Einsatz von DiGA wird in medizinischen Leitlinien bisher kaum berücksichtigt, obwohl dies zur Förderung und Etablierung im Versorgungsprozess notwendig wäre.

## Footnotes

1 Auffindbarkeit, Zugänglichkeit, Interoperabilität, Wiederverwendbarkeit.

2 Kommunikation im Medizinwesen (KIM), Telematikinfrastruktur-Messenger (TIM).

3 DIGIOP: „Digital-gestützte Versorgungsabläufe möglich machen: Interoperabilität zwischen DiGA, Hilfsmitteln und ePA zukunftsoffen umsetzen“.

4 GAIA-X: Europäisches Technologieprojekt zur Schaffung einer vernetzten Dateninfrastruktur nach europäischen Standards zur Förderung von Datenschutz, Innovation und Kooperation.
